# Family and Fertility: Kin Influence on the Progression to a Second Birth in the British Household Panel Study

**DOI:** 10.1371/journal.pone.0056941

**Published:** 2013-03-13

**Authors:** Paul Mathews, Rebecca Sear

**Affiliations:** 1 Institute of Social and Economic Research, University of Essex, Colchester, United Kingdom; 2 Department of Population Health, London School of Hygiene and Tropical Medicine, London, United Kingdom; Durham University, United Kingdom

## Abstract

Particular features of human female life history, such as short birth intervals and the early cessation of female reproduction (menopause), are argued to be evidence that humans are ‘cooperative breeders’, with a reproductive strategy adapted to conditions where mothers receive substantial assistance in childraising. Evolutionary anthropologists have so far largely focussed on measuring the influence of kin on reproduction in natural fertility populations. Here we look at the effect in a present-day low-fertility population, by analysing whether kin affect parity progression in the British Household Panel Study. Two explanatory variables related to kin influence significantly increase the odds of a female having a second birth: i) having relatives who provide childcare and ii) having a larger number of frequently contacted and emotionally close relatives. Both effects were measured subject to numerous socio-economic controls and appear to be independent of one another. We therefore conclude that kin may influence the progression to a second birth. This influence is possibly due to two proximate mechanisms: kin priming through communication and kin assistance with childcare.

## Introduction

Evolutionary fitness is measured by the relative frequency of one's genes in subsequent generations. An individual can gain fitness through another organism's successful reproduction, but only if they share genes. The greater the relatedness the more the benefits of cooperation can outweigh the costs. Fitness so defined includes the successful reproduction of both the individual *and* their relatives into a total ‘inclusive’ measure [1]. Inclusive fitness theory has been vital in understanding the reproductive behaviour of numerous species, most notably the social insects but also various birds and mammals, where the role of relatives is so important that the reproductive strategy of the species is described as ‘cooperative breeding’ [2], [3]. Hrdy [4] has argued that humans should also be classified as cooperative breeders and empirical evidence suggests that in high-fertility resource-poor settings the presence of kin is beneficial for fitness outcomes, namely child survival [5] and fertility [6], [7] (though also see Strassmann [8]).

Here we examine the extent to which humans in a resource-rich contemporary environment have patterns of reproductive behaviour that fit this cooperative breeding hypothesis. It should be noted that reproduction in such settings is often considered to be ‘maladaptive’ because the abundant resources available are not converted into more offspring. There have been many attempts at reconciling evolutionary theory and low levels of fertility [9]. Two important contributions to this debate by Turke [10] and Newson et al [11] stress that the weakening of kin ties may lead to low levels of fertility, though via different proximate mechanisms: respectively, kin assistance and kin priming. Whilst these theories set out to explain fertility variance at the population level, here we apply them to explaining within population variance. We will discuss in detail the mechanisms proposed over the next two sections.

We have found a limited number of empirical tests of kin influence on childbearing in low-fertility settings. What evidence that there is presents a mixed picture. Some previous studies [12]–[14] have reported that kin have a pro-natal influence on women's fertility, using a diverse array of measures of both kin and fertility. Childcare provided by relatives has also been shown to increase fertility intentions [15]. However, Hank and Kreyenfeld [16] found no effect of informal or kin-provided childcare on second birth progression in Germany.

Using the same dataset we analyse here, we have previously shown [17] that having a larger number of kin in a woman's close social network reduces her age at first birth. Different considerations may apply to the decision to have a subsequent birth, so in this paper we test whether kin effects are replicated for another fertility measure: the transition to a second birth. There are also different considerations which apply to the analysis of first versus subsequent births. There is a relatively short period of time during which subsequent births are likely to occur, which is known as the ‘childbearing window’ [18], [19], whereas women may have their first birth anytime between their teens and early 40s. Here we focus on this ‘childbearing window’. We also exclude transitions to 3^rd^ and later births because there were too few of these births in our dataset to allow separate analyses. We chose not to analyse the transitions to 2^nd^, 3^rd^ and later births simultaneously as the decision to continue to 3^rd^ and higher order births in a low fertility population is taken only by a small (and likely unusual) sub-section of the population. Therefore including these transitions may introduce noise into our analysis.

We investigate two potential proximate mechanisms by which kin could exert a pro-natal influence. First, resources and practical assistance from relatives may reduce the cost of having children and thus encourage further childbearing [10]. Secondly, communications with kin could have the effect of ‘priming’ pro-natalism [11]. These mechanisms are not mutually exclusive but they can theoretically work independently of each other.

### Kin assistance

Kin provide important assistance in resource-poor and natural fertility societies [20]–[22]. In these environments relatives can directly influence fertility by enhancing a female's health and thus her fecundity. Kin who provide resources directly to the child could also allow earlier weaning and cessation of lactational amenorrhea, which may in turn lead to shorter birth intervals and higher overall fertility. Kin assistance directed towards mothers and offspring is the mechanism given for how non-human cooperative breeders enhance the reproductive success of their relatives [2], [3].

Kin assistance that helps childbearing is not confined to natural fertility populations. In resource-rich societies relatives can and do provide important resources, in the form of finances or time (i.e. spending time looking after their relatives' children), both of which could reduce the costs of reproduction. Human offspring require extraordinarily high levels of investment from others, normally their parents [23], [24]. Turke [10] argues that in societies where kin provide high levels of assistance this reduces the constraints on parents' childbearing and thus leads to high fertility. Where kin support is weak then the costs are borne more by the parents and they therefore have fewer children. As noted earlier Turke's theory was put forward at a macro-level to explain population level differences in fertility but it is equally plausible that at the individual within-population level those with less kin assistance will have fewer children. For kin assistance to be useful in explaining fertility patterns it must be substantial but not universal. Is this the case in contemporary Britain?

Here we will divide kin assistance into two forms: (i) financial and (ii) childcare. In terms of financial assistance from relatives, Attias-Donfut et al. [25] have shown that in contemporary Europe there are substantial financial transfers between family members. Focusing on grandparents, virtually all the families in the UK's Millennium Cohort Study reported some financial assistance from at least one grandparent [26]. Two other British datasets [27], [28] showed that around 50% of grandparents report ‘regularly’ providing financial support. There is some evidence to suggest that the level of kin's financial assistance has increased over recent years. In 1997 10% of first-time house-buyers under 30 required informal assistance to purchase their property, but by 2005 nearly 50% had assistance from ‘family or friends’[29] (presumably more the former than the latter).

In terms of kin providing childcare, the literature broadly shows that in the UK and other economically developed countries ‘informal’ childcare (childcare that is not purchased) is substantial, though not universal. For example Fergusson et al.'s [30] analysis indicates that around 45% of young children were cared for by a grandparent and 10–15% by ‘other relatives’. These patterns are also seen in other studies where the provision of childcare and other resources by relatives, particularly the child's grandparents, is significant but varies considerably between sub-groups of the population [25], [28], [31]–[39].

### Kin Priming

Kin priming is a potentially independent proximate mechanism that allows relatives to influence the fertility of their relatives. This idea is based on the recent work of Newson et al. [11] and a key component is that it is communication, rather than resources, that influences fertility. From an inclusive fitness perspective it will often be adaptive for relatives to provide information that encourages or primes individuals towards pro-natal sentiments and thus raises fertility. It should be noted that such priming of relatives may, or may not, be overt or conscious. On the one hand parents could explicitly attempt to persuade their adult children to provide them with grandchildren. It could also be much more subtle, with relatives raising conversation topics pertinent to childbearing, leading conversations to more pro-natal conclusions as well as indirectly encouraging decisions (regarding partnership, housing, employment etc) that are more conducive to childbearing. Such priming could in turn lead to the formation of reproductive norms that are more or less fitness maximising. For example, the acceptability of voluntary childlessness may be greater in non-kin orientated social groups than in kin orientated groups.

Empirical evidence on whether kin do communicate more pro-natal messages is currently rather limited. Newson et al. [40] found in role playing experiments those playing the ‘mother’ role provided more fitness maximising messages than those playing a ‘friend’ role. Keim et al. [41] found in qualitative social network analysis that kin seemed to provide social pressure on respondents to have children. Axinn et al. [42] provide some evidence for direct conscious persuasion as a mother's preferences for grandchildren were correlated with her adult children's preferences for children. Waynforth, while not directly examining the content of communication between kin, found that emotional closeness and frequency of contact with maternal grandparents increased the likelihood of birth for UK women [14].

Kin priming does not require the transfer of resources, but it does require communication between relatives. To help explain variance in second birth transitions the amount of communication with kin in contemporary Britain must, like kin assistance, be substantial but also variable. This seems to be the case. Hawkes and Joshi [26] found that 65% of new mothers saw their mother ‘at least’ weekly and one-fifth ‘daily’. It has also been estimated that 20% of British adults contact a sibling ‘at least several times a week’ [43]. However, frequency of contact with kin varies with numerous factors such as age [31] occupation and education [44], geographic proximity [45], ethnicity [46] and local area deprivation [27], [47].

In summary, there may be two proximate mechanisms, kin assistance and priming, through which kin could influence a female's transition to second birth. In this analysis we will attempt to distinguish between the effects of these two mechanisms. Our *a priori* prediction is that both kin influence indicators will have a pro-natal effect on fertility.

## Methods

Our data come from six waves of the British Household Panel Study (BHPS) conducted between 1992 and 2003. The BHPS is a multipurpose longitudinal dataset that is broadly representative of the British population, though results should not be taken as strictly representative of the population of Great Britain as it was not possible to fit a satisfactory weighting (full documentation of question wording, data collection methods etc is available at http://www.iser.essex.ac.uk/survey/bhps).

We restricted our analysis to females as the household clustering of the survey means that male and female respondents were often partners and so their fertility was highly correlated. There are also well known reporting problems with male fertility [48]. We used discrete-time event history analysis with the dependent ‘event’ being a second birth [49], [50]. This technique divides a respondent's recorded time into spells, which in this case last for two years. Surveys were conducted annually but not all variables were collected during every survey. For example, the key kin priming indicators were only collected every other year. A respondent entered the dataset once her first child was born. She exited the dataset in four ways i) her second child was born, ii) she reached 45 years of age, iii) she reached 10 years since first birth without an additional birth (we removed observations after this point as the probability of ever having a second birth becomes very low), or iv) she reached the end of the data collection period without having a second birth (i.e. she was right-censored). Multivariate binary logistic regression models were then fitted for predictors of the occurrence of a second birth. Event history analysis has long been regarded as a good technique for understanding birth progressions [51] since it allows for both time-varying covariates and censorship in the dataset. An assumption of this technique is that the covariates have proportional hazards across the time period under analysis. We broadly confirmed this assumption is valid for the models presented here. This and the other sensitivity analyses mentioned but not shown are available on request from the authors.

### Dependent variable

The dependent event variable used was whether or not a second birth occurred within a window of 9–27 months after the respondent was interviewed. A nine month lag was used to ensure that respondents were not pregnant at the time they reported on the indicators of kin priming and kin assistance. This controlled for a potential confounding effect whereby relatives may increase childcare or contact in response to pregnancy. Respondents who were pregnant (i.e. had a birth within nine months of the interview) were not included in the analysis. A cut-off of 27 months was used since relatives may change their contact and assistance over time and it would be inappropriate to attribute the influence of these indicators too far into the future. We tested the sensitivity of the analysis by changing the lag for the measurement of the event (second birth) to either 9–18 or 18–27 months after the interview. The results stayed broadly similar.

### Explanatory variables

Our explanatory variables explored kin priming and kin assistance as proximate mechanisms through which kin can influence the transition to a second birth.

Kin priming indicators were constructed from responses to a battery of questions asked in alternate waves of the study about the three individuals the respondent felt *‘closest’* to outside of their household. We will describe these individuals as ‘emotionally close’ to the respondent. Two key variables were constructed from the responses to this battery i) the number of relatives who were emotionally close and ii) the number of relatives who were emotionally close relatives and were also in frequently contact (defined as those who reported contacting the respondent *‘most days’* by either *‘visiting, writing or by telephone.’*)

We tested the robustness of the findings for the kin priming variables by running the model again using different operationalisations. First, we used dichotomised versions of the variables i.e. whether the respondent had *any* emotionally close relatives. Secondly, we decreased the threshold for the frequency of contact to *‘at least once a week’*. Both of these changes produced similar results though there was a decrease in the statistical significance level.

Two additional explanatory variables were constructed from this battery of questions on emotionally close individuals. We created separate variables which coded for whether a specific category of relative (e.g. mother, sister) was listed as one of these individuals, to determine if a particular relative was driving any overall effect. We also looked at emotionally close relatives' geographic proximity. We created a set of variables which combined the geographical proximity and frequency of contact for each emotionally close relative. The categories were whether the respondent had an emotionally close relative: (i) within 50 miles of respondent and frequently contacted, (ii) within 50 miles but infrequently contacted and (iii) over 50 miles from respondent (there were only 19 occasions when respondents lived over 50 miles away and also frequently contacted an emotionally close relative, so we made a single group for occasions when the relative lived over 50 miles away). Some forms of kin assistance, such as childcare and help with day-to-day activities can more easily be provided if relatives live in close proximity. Conversely kin priming can occur wherever kin live, as long as they are in contact with the respondent. So if kin assistance is the more important mechanism, then relatives who are geographically distant should have a weaker influence on the transition to a second birth. If kin priming is the more important mechanism, then frequently contacted relatives, regardless of their geographic location, should have the greater pro-natal effect. Unfortunately the questions on specific relatives and geographic proximity were not asked in one wave of the BHPS (Wave F 1996-1997) and so we have one sixth fewer observations for this part of the analysis.

We looked for indicators of kin assistance. We were unable to construct a variable based upon whether kin provided financial assistance to the respondent. Surprisingly there were only 11 occasions (less than 1%) where the respondent reported receiving a payment from a non-household family member. Compared to the levels of kin financial assistance found in the papers discussed earlier, this value for mothers with young children seems implausibly low [46] [25]. We suspect this is simply measurement error on the part of the BHPS.

However, many women in the BHPS did report receiving assistance with childcare from relatives. Respondents were asked “which of the following best describes the way you arrange for your children aged 12 or under to be looked after while you are at work?” The interviewer then recorded up to three responses from a list of eleven potential childcare categories. We created three dummy variables for different childcare types. A kin assistance explanatory variable was constructed with ‘1’ if the respondent mentioned ‘a relative’ as one of these three forms of childcare and ‘0’ if a relative was not mentioned. A second dummy variable for ‘formal childcare’ was constructed if the respondent mentioned ‘at school’, ‘nanny/mother help’, ‘day nursery’ or ‘childminder’. The final ‘other childcare’ dummy variable combined instances of the respondent mentioning ‘spouse or partner’, ‘friend’ or ‘other’. These additional childcare variables were necessary to control for the effect of women using childcare per se. The reference category was set as the occasions when the respondent did not mention anyone else providing childcare. Potential weaknesses of this childcare question are that it does not capture the full extent of childcare and there could be variation in how respondents interpreted the notion of ‘work’. We return to this issue in the discussion section. We did not distinguish between whether the type of childcare was mentioned first, second or third, or whether it was mentioned in conjunction with other methods (but there were only a limited number of occasions when the respondent mentioned more than one type of childcare).

Respondents were only included within the dataset when they were present in a wave of data collection containing the battery of questions about individuals who were emotionally close to the respondent. If respondents were missing for one wave of data collection they were still eligible for inclusion in later waves subject to the usual constraints, such as not being pregnant with a second child.

### Control variables

There are numerous factors that influence childcare provision and kin contact and these same factors could also confound any observed association with the second birth transition. First, three types of time measurement are included in all of the models. The event history analysis is based on the number of months since the respondent's first birth. Time since first birth will be associated with the likelihood of second birth due to the ‘childbearing window’ discussed earlier. It also captures the first born child's age and this will correlate with the nature of childcare, as older children are eligible for (pre)schooling. Secondly, the respondent's age is included, as a female7s age is associated with both her fecundity and the nature of her family and friend contacts [52]. Finally all models include dummy variables for the wave (year) of the survey to take into account calendar time, though their effect is consistently non-significant and so they are not reported.

To determine whether it was an individual's general level of social attachment, rather than interactions with kin, that might be causing any observed correlations we included a measure of the number of frequently contacted emotionally close individuals (regardless of whether they were related). We control for socio-economic confounders. Female employment increases childcare demand, and at the macro-level there is a ‘U’ shaped association with fertility [53]. The extent of employment is included as the number of reported hours the respondent is in paid employment (including overtime). We also control for education, as it is often shown to be associated with birth interval length [18] and is also associated with kin interactions [52] and childcare use [30], [34] though see [37]. Education is included as a time-varying covariate (though in reality very few of the respondents obtained additional qualifications during the study period. For example less than 2% of those without a degree obtained one during this period). We also included the respondent's income and her proportion of household income. Income will influence the capacity to purchase formal childcare in place of informal childcare, and the opportunity costs of leaving the labour force [54]. Family networks and childcare practices also differ between ethnic-cultural groupings [55] and so does fertility [56], [57]. We therefore included non-time varying controls for religious organisation membership and non-white ethnicity.

We included several variables on household composition, as our kin priming variables only include non-household members. For example, having a resident partner will clearly influence fertility and it also influences grandparental childcare involvement [28]. We also controlled for the sex of the first born child. This can influence numerous parental behaviours, including fertility and childcare [58]. We included a control variable for the focal women's sibship size as this would influence the number of non-household relatives who were available for inclusion within our measure of emotionally close relatives, and there is also a known correlation between the fertility of parents and offspring [59]. Scotland and Wales were over sampled in later waves of the BHPS, so we included dummy variables for both countries in the models.

We also tested for significant interactions between the explanatory variables and all the socio-demographic control variables. This was because the pro-natal (or anti-natal) influence of relatives on fertility could be limited to particular sub-groups in the populations and the observed aggregate effects could mask both pro-natal and anti-natal effects. However, we did not find any consistent interactions between the socio-demographic controls and the explanatory variables that were statistically significant at the 5% level. Finally we checked for non-linear effects for each of the continuous variables and a quadratic term was included in the final model for age to control for non-linearity. No other non-linear effects were found to be significant.

All analysis was conducted using Stata. Data collection, storage and use is administered by the Institute of Social and Economic Research, University of Essex in accordance with the Ethical Guidelines of the Social Research Association.

## Results

Our final dataset contains information from 594 females who had had a first birth. The earliest age at interview in the dataset came from a female who was 17 and the oldest 44. These women contribute 1115 spells of data (i.e. observations). 242 second births were captured in the 9–27 month threshold after the interview meaning that 21.7% of spells ended in a second birth. Table 1 shows the descriptive statistics for the explanatory variables. It also shows the volatility of the time varying covariates for respondents who contributed data in two or more waves, by showing the percentage of individuals who ever changed value for each covariate. Descriptive statistics for the categorical and continuous control variables are shown in Table 2 and Table 3.

**Table 1:** Descriptive statistics of explanatory variables.

Variable	Number of spells	% of spells	% spells followed by a 2^nd^ birth	% individuals who ever change value^1^
No. of emo. close relatives (used as a continuous variable)				
0	439	39.37	18.68	31.07
1	445	39.91	22.02	41.79
2 (or 3)	231	20.72	26.84	23.21
No. of emo. close relatives freq. contacted (used as a continuous variable)				
0	711	63.77	19.69	37.50
1	291	26.10	24.40	40.00
2 (or 3)	113	10.13	27.43	17.14
Childcare variables				
Does not use childcare (reference)	424	38.03	21.23	33.93
Childcare provided by a relative	275	24.66	25.09	31.43
Formal childcare used	396	35.52	21.72	41.43
Other form of childcare used	251	22.51	18.33	37.14
Specific emo. close relative mentioned below				
A parent	280	30.53	26.43	21.07
Her mother	267	29.12	27.34	21.07
Her father	40	4.36	27.50	4.64
A sibling	281	30.64	23.84	19.29
A sister	252	27.48	24.21	16.79
A brother	36	3.93	27.78	4.29
An ‘other relative’	96	10.47	23.96	10.00
A relative is emo. close but lives over 50 miles away	105	11.45	30.48	11.79
A relative is emo. close, lives within 50 miles but infreq. contacted	199	21.70	21.61	25.36
A relative is emo. close, lives within 50 miles and freq. contacted	299	32.61	25.08	28.57

1 If the individual provided information in two or more waves

**Table 2:** Descriptive statistics of categorical control variables.

	*Number of spells*	*% of spells*	*% spells followed by a 2^nd^ birth*	*% who ever change value* 2
No. of freq. contacted emo. close friends (used as a continuous variable)				
0	353	31.66	24.65	41.43
1	374	33.54	19.79	54.29
2	251	22.51	22.31	43.57
3	137	12.29	18.25	26.43
First born child is female	546	48.97	22.34	n/a
No. of siblings (used as a continuous variable)				
0	259	23.23	18.53	n/a
1	334	29.96	24.85	n/a
2	264	23.68	21.21	n/a
3	149	13.36	22.82	n/a
4	47	4.22	25.53	n/a
5 or more	62	5.56	14.52	n/a
Sibship size: Missing^3^	133	11.93	15.79	n/a
Education: University or equivalent	199	17.85	31.66	1.79
Education: A level or equivalent	237	21.26	21.94	4.64
Education: Less than A level or missing (reference)	670	60.09	18.96	2.86
Ever member of religious organisation	190	17.04	27.89	n/a
Non-white ethnicity	43	3.86	18.60	n/a
Lives in England (reference)	963	86.37	21.70	0.36
Lives in Scotland	99	8.88	21.21	0
Lives in Wales	53	4.75	22.64	0.36
Household contains				
No other adults (reference)	229	20.54	10.92	20.00
A partner	801	71.84	25.59	21.43
A sibling	48	4.30	14.58	4.64
A parent	89	7.98	12.36	9.64
Another adult	23	2.06	21.74	3.93

2 If the individual provided information in two or more waves

3 Missing variable coded as 1 if information is unavailable, and 0 if information is available

**Table 3:** Descriptive statistics of continuous variables.

	Mean	Standard Deviation
Time employed, including overtime (hours)	18.66	17.28
Individual annual income (£1000s in 2005 equivalent purchasing value, adjusted by Consumer Price Index)	9.99	8.84
Percentage household income earned by respondent	46.13	33.75
Time since 1st birth (months)	43.88	33.76
Age (years)	30.43	6.07
No. of individuals in the household (capped at 6)	2.97	0.76

Column three of Table 1 shows the percentage of spells that end with a second birth 9–27 months after measurement for each of the categorical variables. The bivariate association is in the expected direction for both the kin assistance and kin priming indicators. As the number of emotionally close relatives increases so does the percentage of spells ending in a second birth (chi-square association significance p = 0.05). The same association is seen for those relatives who are also frequently contacted, though it is only marginally significant (chi-square association significance p = 0.07). Where a relative provides childcare, 25% of spells end in a second birth, which is slightly higher than when the respondent does not mention receiving childcare (21%), though this association falls outside of conventional statistical significant (chi-square association significance p = 0.17).

Table 4 shows the results for the multivariate discrete-time event history models with the first three explanatory variables included separately (model one - number of emotionally close relatives, model two - number of frequently contacted emotionally close relatives and model three - whether a relative provided childcare). The results are presented as odds ratios, whereby a value greater than one indicates that this variable increases the odds of a second birth occurring 9–27 months after the interview compared to the reference category (controlling for all other variables), whilst a value less than one indicates that this variable decreases the odds of a second birth. We included all the theoretically relevant control variables in each model though it should be noted that many do not significantly predict the occurrence of a second birth. In a ‘best fitting model’ produced from backwards model selection, the explanatory variable's effects are essentially the same as those displayed here.

**Table 4:** Multivariate results - explanatory variables main effects.

	Model 1		Model 2		Model 3	
	OR	*p*	OR	*p*	OR	*P*
No. of emo. close relatives	1.140	0.233				
No. of freq. contacted emo. close friends	1.025	0.773				
No. of emo. close relatives freq. contacted			1.262**	0.045		
Childcare provided by a relative (ref: Does not use childcare)					1.604**	0.018
Formal childcare used (ref: Does not use childcare)					1.496*	0.051
Other form of childcare used (ref: Does not use childcare)					0.733	0.143
First born child is female	1.118	0.486	1.117	0.492	1.119	0.486
Time employed, including overtime (hours)	0.997	0.655	0.997	0.641	0.990	0.169
Individual annual income (£1000s 2005 CPI)	1.010	0.478	1.010	0.454	1.009	0.522
Percentage household income earned by respondent	0.993	0.137	0.993	0.128	0.993	0.156
No. siblings	0.976	0.722	0.983	0.793	0.984	0.806
No. siblings: Missing	0.643	0.157	0.652	0.169	0.666	0.195
Education l: University or equivalent	1.747**	0.012	1.788***	0.009	1.731**	0.014
Education: A level or equivalent	0.877	0.529	0.891	0.58	0.857	0.46
Ever member of religious organisation	1.423*	0.089	1.424*	0.088	1.434*	0.084
Non-white ethnicity	1.081	0.866	1.093	0.847	1.101	0.835
lLves in Scotland (ref England)	1.041	0.888	1.036	0.9	0.991	0.974
Lives in Wales (ref England)	1.149	0.709	1.144	0.716	1.090	0.816
Household contains (ref Lives Alone)						
A partner	1.978	0.158	1.991	0.155	2.125	0.122
A sibling	2.315	0.238	2.348	0.232	2.465	0.21
A parent	0.735	0.661	0.713	0.629	0.630	0.514
Another adult	1.752	0.385	1.709	0.406	1.877	0.336
No. of individuals in the household (capped at 6)	0.682	0.167	0.688	0.179	0.680	0.174
Time since 1st birth (months)	0.984***	0	0.984***	0	0.983***	0
Age (years)	1.701***	0.001	1.687***	0.001	1.679***	0.001
Age (years squared)	0.990***	0	0.990***	0	0.990***	0
Pseudo – r squared	*0.135*		*0.137*		*0.143*	

Controlling for wave of data collection (non-significant in all models)

*** p<0.01 **p<0.05 *p<0.1

Model one shows that once our control variables have been added the number of emotionally close relatives no longer significantly predicts the occurrence of a second birth. On the other hand, model two shows the number of emotionally close relatives who are frequently contacted does significantly increase the odds of a second birth. Model three shows that once the full set of controls is included when kin provided childcare statistically significantly increase the odds of a second birth. These variables all relate to relatives outside the household. In none of these models do the household composition variables significantly predict the occurrence of a second birth (i.e. having a parent or sibling in the same household).

Table 5 shows a summary of the remaining models. This table only contains the results of the explanatory variables though the full set of control variables was used in all cases (we have only shown the explanatory variables because the controls provided very similar results to those already seen.) Models four and five, which include main effects for both types of explanatory variable (kin assistance and kin priming) included together in the same model. The results remain very similar and the fit of the model (pseudo r-squared) increased only very slightly (by 0.002 in Model 4 and 0.003 in Model 5), though the effect for frequently contacted relatives becomes significant only at the 10% level. It is quite possible that there is a correlation between the number of emotionally close relatives a respondent reports and whether a relative provides childcare, since relatives who provide childcare may well also be emotionally close to the respondent. Table 6 shows the bivariate association between the explanatory variables. It shows that there is little association between the number of emotionally close relatives a respondent reports and whether a relative provides childcare (Kendall's tau−b = −0.02, p = 0.47). However there is a significant, positive relationship between the number of frequently contacted emotionally close relatives and relative-provided childcare (Kendal's tau−b = 0.08, p = 0.01). We tested interaction terms between our relative-provided childcare variable and both the (i) number of emotionally close relatives and (ii) number of frequently contacted and emotionally close relatives. Neither interaction term was significant, with a very small effect size for the interaction with frequently contacted relatives. This means that there is not a significant multiplicative effect of both kin childcare provision and frequently contacted emotionally close relatives.

**Table 5:** Multivariate results – explanatory variables in a combined model and other explanatory variables.

Model	Explanatory variable	OR	*p*
4	No. of emo. close relatives	1.138	0.244
	No. of freq. contacted emo. close friends	1.009	0.921
	Childcare provided by a relative	1.598**	0.019
	Formal childcare used	1.514**	0.045
	Other form of childcare used	0.747	0.172
5	No. of emo. close relatives freq. contacted	1.231*	0.08
	Childcare provided by a relative	1.545**	0.03
	Formal childcare used	1.535**	0.039
	Other form of childcare use	0.760	0.197
Reference categories for above models (4–5): *Does not describe a relative as emotional close and does not use childcare*			
**If the specified relative is emotionally close**			
6	A parent	1.133	0.500
7	Mother	1.207	0.312
8	Father	1.455	0.352
9	A sibling	1.226	0.284
10	A sister	1.274	0.215
11	A brother	1.275	0.561
12	An ‘other relative’	1.061	0.831
Reference category for above models (6–12): *Does not describe that relative as emotional close*			
**Geographic distance, freq. of contact and emotional close relatives**			
13	A relative is emo. close but lives over 50 miles away	1.330	0.278
	A relative is emo. close, lives within 50 miles but infreq. contacted	0.949	0.808
	A relative is emo. close, lives within 50 miles and freq. contacted	1.291	0.178
Reference category for above model (13): *Does not describe any relatives as emotional close*			

Each model is a separate multivariate regression controlling for the same control variables as used in Models 1–5 but only the results for the explanatory variable are displayed above. Each model included a single explanatory variable(s). All other control variables were included in all of the models. Specifically the control variables used were: First born child is female, Time employed includes overtime, Individual's annual income, Percentage of household income earned by respondent, Sibship size, Education level, Religiosity, Ethnicity, Respondent lives in Scotland or Wales, Household composition, Time since 1st birth, Age (with quadratic term) and Wave of data collection

Note all the above models (6–13) exclude Wave 3 so n = 917. However, when models 1–5 were run on the subset of data excluding Wave 3 the results were very similar to those shown.

**Table 6:** Bivariate relationship between number of family in the friendship group and childcare provision by relatives.

		Childcare provided by a relative
No. of emo. close relatives		
0	N	118
	*% (i.e. of those with no emo.close relatives who have childcare provided by a relative)*	*26.88*
1	N	96
	*%*	*21.57*
2/3	N	61
	*%*	*26.41*
No. of emo. close relatives freq. contacted		
0	N	157
	*%*	*22.08*
1	N	82
	*%*	*28.18*
2/3	N	36
	*%*	*31.86*

Finally, we also analysed two other sets of explanatory variables, whether a specific relative was described as emotionally close and the geographic distance of the emotionally close relative. A summary of the results of the multivariate models are also set out in Table 5. The two most commonly cited relatives were mothers, included in 29% of spells, and sisters, included in 27%. We included the presence of a specific relative as the sole explanatory variable in multivariate models six to twelve and in all cases the presence of the specific relative was in the anticipated direction (it increased the odds of a second birth), though the effect was not statistically significant. This suggests that the aggregate effect of emotionally close relatives was not driven by the presence of one particular type of relative. Unfortunately the BHPS does not provide information on which relatives are providing childcare, so we were unable to explore this aspect further, though the literature suggests it would largely be the child's grandparents.

We also analysed an interaction between geographic proximity and frequent contact with emotionally close relatives. Model thirteen shows that the geographic proximity variables are not significant. Kin who are geographically closer could more readily provide childcare, so the lack of a geographic difference adds some support to the idea that kin influence is not solely due to assistance with childcare.

## Discussion

These results suggest that kin positively influence the progression from first to second birth in contemporary Britain. A second more tentative conclusion is that both proximate mechanisms, kin assistance and kin priming, are important, given that both being in frequent contact with emotionally close relatives, and having childcare provided by a relative, appeared to be associated with progression to second birth. However, only very cautious conclusions on the relative importance of the proximate mechanisms should be drawn. First, relatives who help with childcare can also encourage and prime the respondents to have an additional child. We are not able to match the relatives in the two indicators together but there is a significant bivariate association between a respondent reporting frequent contact with emotionally close relatives and a respondent reporting that her first born child is looked after by a relative, so it seems plausible that those relatives who provide childcare are also those who are emotionally close to the respondent. Secondly, the temporal relationship between kin priming and kin assistance is unclear. Are relatives in close social proximity simply more likely to undertake childcare, or does the provision of childcare bring relatives closer together? We are unable to distinguish between these two possibilities in this analysis of second birth, but we note that our previous analysis of progression to first birth found that being emotionally close to one's relatives increased the odds of birth even before relatives are able to undertake childcare for the respondent [17].

In this analysis we are using indicators of kin priming and kin assistance, and there are limitations in the extent that these concepts are fully captured. Kin priming could be induced by relatives other than the three the respondent considers particularly close at that point in time. Kin assistance can be much more substantial than simply the provision of childcare whilst the respondent is ‘at work’. An additional limitation is that we are not able to properly identify which kin are important. Our dataset has relatively crude, composite kin indicators. ‘Kin’ are not a homogenous entity, and our indictors may mask the effects of relatives varying in their motivation and capacity to influence fertility. We do not know who is providing childcare other than that they are ‘a relative’. When we tested each specific emotionally close relative (mother, sisters etc) with progression to second birth, none were significant, though all had positive effects, and non-significance could simply be due to an insufficient number of observations.

Both our kin variables are only measured in the immediate environment. Many species, and particularly humans, have ‘developmental plasticity’ whereby information from earlier environments shapes the individual organism's physiological and psychological development [60], [61]. During the developmental period some individuals may be more influenced by non-kin, who may instil values towards a life course that is not fitness maximising, or kin, who may induce ‘family values’ that are more fitness maximising. The developmental period could be important, but in this study we are only able to look at the components of kin influence in adulthood. It would be interesting for future research to look at a wider array of indicators of kin influence and in particular to also look at *when* kin influence has the greatest impact.

Finally, we have concluded that we find some support for the hypothesis that kin have a direct influence on the fertility. An alternative explanation is that there is some unmeasured common factor which predisposes women both to remain close to their kin, and to have relatively high fertility. For example, the associations we have found could be confounded by unmeasured personality characteristics of the respondents. Nettle [62] has argued that human personality diversity, as represented in Costa and McCrae's [63] five factor (Neuroticism, Extraversion, Openness, Agreeableness and Conscientiousness) model has an ultimate adaptive function. It is ultimately adaptive for multiple personality strategies to co-exist in a population, as the optimal fitness maximising personality will depend upon the local ecology (resource availability, population density etc) and the frequency of competitors’ personalities [64]. There is some evidence that personality components do influence the frequency of contact with relatives [65]. By definition low levels of Openness or Extraversion are likely to limit interactions with ‘new’ individuals (who are liable to be non-kin). Some personality traits do seem to be associated with some aspects of fertility, although these links tend to vary cross-culturally. For example, neuroticism is positively correlated with fertility in some populations [66], [67] and negatively in other [68].

We were unable to control for personality in our analysis. It was measured in the BHPS, but only many years after most of our observations. Personality can be relatively stable across the life course, but there are circumstances, such as major life events, that are shown to be associated with changes in personality [69], [70]. Indeed Jokela et al. [68] and Srivastava et al. [71] have shown that parenthood may influence personality, increasing Agreeableness and Emotionality scores. Additionally, personality development may be subject to kin influence. Parenting styles influence children's personality development [72]. So personality may not be entirely separable from kin influence.

The BHPS was designed as a multipurpose survey, without our research question in mind. Many of the above limitations are products of using data of this nature. Nevertheless the BHPS is a good dataset in many other ways, providing broadly representative and longitudinal information. It has allowed us to show that there is a significant association between kin influence indicators and fertility, even after numerous socio-economic controls. We therefore argue that the key conclusion from these results is that in a resource-rich low-fertility setting the cooperative breeding hypothesis helps predict the pattern of observed fertility behaviour. A mother with a young child who scores highly on indicators of kin assistance and kin priming has a higher propensity in a given period of time following her interview to have a second birth. Kin can influence fitness in a setting where fertility is low and child survival is extremely high. Fitness is a relative measure, comparative to an organism's competitors. Indeed because children are discrete units, each additional child in a low fertility environment represents a proportionally higher increase in reproductive success than in a high fertility society. This paper complements our earlier work which showed the positive influence of kin on the transition to first births in the same low fertility population.

Social scientists have long been interested in fertility and family structure [73], [74]. However there has been relatively little recent research on if and how family influence fertility in resource-rich societies. Our results demonstrate the utility of a cooperative breeding perspective when seeking to understand contemporary family and fertility behaviour.
